# Developing Proprioceptive Countermeasures to Mitigate Postural and Locomotor Control Deficits After Long-Duration Spaceflight

**DOI:** 10.3389/fnsys.2021.658985

**Published:** 2021-04-27

**Authors:** Timothy R. Macaulay, Brian T. Peters, Scott J. Wood, Gilles R. Clément, Lars Oddsson, Jacob J. Bloomberg

**Affiliations:** ^1^KBR, Houston, TX, United States; ^2^NASA Johnson Space Center, Houston, TX, United States; ^3^RxFunction Inc., Eden Prairie, MN, United States; ^4^Department of Rehabilitation Medicine, University of Minnesota, Minneapolis, MN, United States; ^5^Recaniti School for Community Health Professions, Ben Gurion University of the Negev, Beersheba, Israel

**Keywords:** microgravity, balance, Mars, sensorimotor, weightlessness, bed rest

## Abstract

Astronauts experience post-flight disturbances in postural and locomotor control due to sensorimotor adaptations during spaceflight. These alterations may have adverse consequences if a rapid egress is required after landing. Although current exercise protocols can effectively mitigate cardiovascular and muscular deconditioning, the benefits to post-flight sensorimotor dysfunction are limited. Furthermore, some exercise capabilities like treadmill running are currently not feasible on exploration spaceflight vehicles. Thus, new in-flight operational countermeasures are needed to mitigate postural and locomotor control deficits after exploration missions. Data from spaceflight and from analog studies collectively suggest that body unloading decreases the utilization of proprioceptive input, and this adaptation strongly contributes to balance dysfunction after spaceflight. For example, on return to Earth, an astronaut’s vestibular input may be compromised by adaptation to microgravity, but their proprioceptive input is compromised by body unloading. Since proprioceptive and tactile input are important for maintaining postural control, keeping these systems tuned to respond to upright balance challenges during flight may improve functional task performance after flight through dynamic reweighting of sensory input. Novel approaches are needed to compensate for the challenges of balance training in microgravity and must be tested in a body unloading environment such as head down bed rest. Here, we review insights from the literature and provide observations from our laboratory that could inform the development of an in-flight proprioceptive countermeasure.

## Introduction

Crew health and safety are NASA’s top priorities for future exploration missions to the Moon and Mars (Charles and Pietrzyk, 2019). For example, crewmembers must be able to safely perform extravehicular activities (EVAs) and egress vehicles in a variety of landing scenarios. However, NASA’s Human Research Program has identified that decreased mobility due to vestibular and sensorimotor alterations associated with long-duration spaceflight could put the crew at risk in certain landing situations (Bloomberg et al., 2016). This risk is most likely to occur immediately after gravitational transitions such as during potential emergency egress situations when poor mobility could lead to crew injuries (Reschke et al., 1998). Although exercise is an effective countermeasure to mitigate muscular and cardiovascular deconditioning during spaceflight, functional performance decrements on tasks requiring dynamic control of postural stability remain ubiquitous among crewmembers returning to Earth after 6 months missions on board the International Space Station (ISS) (Reschke et al., 2015, 2019). These impairments will presumably be exacerbated after exploration-class missions to Mars, which, based on current propulsion technology, will last about 30 months. Thus, new countermeasures must be developed to physically prepare crewmembers for transitions from microgravity to Mars gravity and from microgravity back to Earth gravity after these very long-duration spaceflights.

Countermeasures for exploration missions must also be accomplished under new constraints. For example, exploration vehicles will impose greater restrictions on exercise capabilities than the ISS. These restrictions include less habitable volume and greater need for vibration isolation and stabilization (Laws et al., 2020). Thus, countermeasure approaches that involve large dynamic hardware, such as an onboard short-arm centrifuge for artificial gravity, are currently not feasible (Clément, 2017). In addition, there are currently no in-flight treadmill designs that fit these onboard constraints. The consequences of excluding an in-flight treadmill are unknown because astronauts have been exercising on in-flight treadmills since Skylab missions in the 1970’s. Although treadmill running onboard the ISS is primarily performed for aerobic conditioning, it is also thought to partially mitigate post-flight sensorimotor dysfunction (English et al., 2020a). Specifically, treadmill running provides a dynamic postural challenge requiring single-limb segmental coordination in response to axial body loading. Treadmill running also allows the central pattern generator to rehearse rhythmic motor outputs that produce periodic sensory input (proprioceptive stretch input, foot tactile input, and cyclical vestibular stimulation) such as that required for the control of terrestrial locomotion. These cumulative stimuli may have an essential sensorimotor training effect for maintaining post-flight locomotion. A study to test the overall effects of substituting treadmill training with other types of aerobic exercise is currently being planned for ISS missions.

The overall purpose of this review is to support the conception and development of countermeasures that address the issue of balance impairments after long-duration spaceflight and are compatible with exploration spaceflight conditions. Rather than describe in detail each of the potential countermeasures that have been proposed, here we elucidate the recent findings that drive current directions. We aim to (a) compare postural and locomotor dysfunction after spaceflight and after spaceflight analogs and describe the effectiveness of exercise for mitigating these dysfunctions; (b) justify the need for and challenges of developing a proprioceptive countermeasure for use in the microgravity environment; and (c) recommend promising modalities for inclusion in an in-flight/in-bed proprioceptive countermeasure.

## Sensorimotor Function After Spaceflight

Central nervous system (CNS) adaptations to spaceflight manifest as a reinterpretation of vestibular and somatosensory inputs (Paloski et al., 1992). On return to Earth, these changes are not suitable for the 1G environment and thus cause sensorimotor dysfunction in standing posture and locomotor control (Reschke et al., 1998). Disruptions after landing include excessive postural sway (Wood et al., 2015), spatial disorientation (Glasauer et al., 1995), alterations in muscle activation variability (Layne et al., 2004), modifications in ankle and knee joint kinematics (Bloomberg and Mulavara, 2003), and alterations in head-trunk coordination (Bloomberg and Mulavara, 2003). Functional tests have been used to assess performance in tasks that will be required for operations after landing on a planetary surface or after the return to Earth. On an obstacle course test requiring locomotor maneuvers that mimic vehicle egress, ISS crewmembers needed approximately 48% more time to complete the test 1 day after returning (R + 1) from spaceflight (average of 185 days in orbit) than they did before takeoff (Mulavara et al., 2010). It then took an average of 15 days for completion time to return to within 95% of preflight levels. In another study, a suite of functional tasks was used to test astronauts after they returned from ISS missions with an average duration of 159 days (Mulavara et al., 2018). On R + 1, subjects exhibited significant impairments in performance on tasks that required whole-body coordination and dynamic control of postural stability. Related sensorimotor impairments were observed in standard tests that evaluate postural equilibrium (the ability to stabilize the body’s center of mass (Horak, 2006) and dynamic gait control. Performance appeared to still be recovering 6 days after landing (Miller et al., 2018).

These recently recorded decrements in post-flight performance were present despite the astronauts performing vigorous exercise as a primary countermeasure while in orbit (English et al., 2020a). Specifically, each ISS crewmember was allowed 90 min per day for resistance training and 60 min per day for aerobic training (includes time for preparation, hardware configuration, and hygiene) with individualized training programs 6 days per week (Loehr et al., 2015). The crewmembers used a suite of aerobic and resistance exercise hardware, including the Advanced Resistive Exercise Device (ARED), the second-generation treadmill (T2), and the Cycle Ergometer with Vibration Isolation System (CEVIS). Compared to their predecessors, these devices enable crewmembers to follow exercise prescriptions that better protect skeletal muscle function and agility (Wood et al., 2011; English et al., 2015). Although these exercise protocols provide greater training stimulus than previous protocols and are critical to attenuate physiological deconditioning (English et al., 2020b), to date, they do not fully protect sensorimotor function after long-duration spaceflight (Reschke et al., 2015, 2019).

## Sensorimotor Function After Spaceflight Analogs

A significant barrier to countermeasure development is the limited opportunity to perform randomized controlled trials in space. Long-duration head down bed rest (HDBR) and dry immersion (DI) are well-accepted analogs of the effects of fluid shifts and axial body-unloading that occur during spaceflight (for reviews, see Pavy-Le Traon et al., 2007; Navasiolava et al., 2011). The primary mechanistic difference between HDBR and DI is the level of support deprivation—HDBR redistributes support loads from the feet to the back and shoulders, while DI eliminates support loads by distributing pressure forces equally around the body surface. Because of this difference, changes to the neuromuscular and sensorimotor systems occur much more rapidly during DI than HDBR, but for the purposes of this review the effects are comparable—including decreased extensor muscle tone, suppressed muscle spindle activation, decreased motor control accuracy, and increased excitability of the soleus spinal reflex. In contrast to the sensory reorganization effects of spaceflight, ground-based HDBR and DI models isolate the impact of body unloading on the somatosensory system while controlling for the effects of Earth’s gravity on the vestibular system (Dupui et al., 1992; Tomilovskaya et al., 2019). This results in impairments in postural stability and locomotor control that parallel those observed after spaceflight despite reduced effects of motion sickness and spatial disorientation (Sayenko et al., 2016; Mulavara et al., 2018). Thus, unloading-induced somatosensory deconditioning plays a significant role in post-flight balance impairments. Since HDBR is better suited for testing dynamic countermeasures such as aerobic and resistance exercise, data from long-duration HDBR countermeasure studies have been used to help investigators understand and mitigate the effects of body unloading (Reschke et al., 2009).

Although not fully protective, in-bed rest exercise typically attenuates balance decrements after HDBR (Saumur et al., 2020). In both 60 and 70 days HDBR studies (Viguier et al., 2009; Koppelmans et al., 2015), subjects who performed horizontal aerobic and resistance exercise during HDBR had similar decrements in obstacle course performance and standing posture control as HDBR control subjects upon re-ambulation. In the 60 days study, exercise subjects recovered static postural control more quickly than the control subjects, but not dynamic postural control (Viguier et al., 2009), while in the 70 days study exercise subjects recovered obstacle course performance faster than the control subjects, but not standing posture control (Koppelmans et al., 2015). In a more recent 60 days HDBR study, a supine jump exercise countermeasure was tested in the form of high intensity interval training (Ritzmann et al., 2018). On BR + 0, the HDBR control subjects who did not exercise had increased postural sway in single-leg stance, reduced locomotor speed concomitant with pathological gait, and increased time to complete a chair rise and walk test, which required at least 8, 8, and 14 days, respectively, to recover to baseline performance. However, the jump exercise group had no changes from baseline in any of these measures, indicating that the jump exercise countermeasure preserved sensorimotor function. Although this finding is promising, such an exercise technique may not be feasible with the expected habitable volume and vibration isolation constraints on an exploration spacecraft (Laws et al., 2020).

Our Functional Task Test (FTT) study compared a 70 days HDBR control group to a corresponding HDBR exercise group that performed a high-intensity integrated aerobic and resistance exercise training prescription using horizontal exercise devices with similar exercise capabilities as those available to ISS astronauts (Mulavara et al., 2018). On BR + 0, the HDBR exercise group had preserved neuromuscular and cardiovascular functions compared to the HDBR control group. However, both HDBR groups had decrements in dynamic control of postural stability that were comparable to performance decrements in ISS astronauts 1 day after return (R + 1) from 6 months of spaceflights. For example, the median time to complete the seat egress and walk test increased by 38% in the HDBR control group and 23% in the HDBR exercise group on BR + 0 and increased 31% in the ISS astronaut group on R + 1. All groups had corresponding impairments in sensorimotor tests that evaluate postural equilibrium and gait control. The HDBR exercise subjects did, however, return to baseline performance more quickly than the HDBR control subjects (Miller et al., 2018). Collectively, these long-duration HDBR studies suggest that the axial body loading and exercise stimulus provided by resistance and aerobic exercise are critical for maintaining muscular and cardiovascular functions and they accelerate sensorimotor recovery; however, they do not fully protect against balance decrements immediately after HDBR. New countermeasures compatible with exploration spaceflight vehicles must be developed to supplement exploration exercise protocols.

## Balance Training in Rehabilitation and Sport

Current balance training interventions on Earth may provide clues for designing in-flight countermeasures. There are two main classes of balance training: reactive and proactive. Reactive balance training protocols involve repeated exposure to instability that mimics balance disturbances. During traditional reactive balance training, subjects attempt to stand or to perform dynamic activities on an unstable surface and use internal feedback to achieve the stability goal (Horak, 2006). Examples include single-leg stance, walking, or jump-to-hold on surfaces such as wobble boards, foam pads, or instrumented tilt platforms (DiStefano et al., 2009). Reactive balance training using moving platform perturbations has been successfully incorporated in clinical rehabilitation protocols for patients with vestibular disorders (Herdman, 2013), and is currently being tested on astronauts before and after spaceflight. Recent studies have examined perturbation-based reactive balance training, which induces unpredictable mechanical perturbations to simulate “real-world” loss of balance. By practicing rapid reactions to postural instability, the goal is to improve the ability to maintain and recover balance in situations that often lead to falls, including slips and trips (Gerards et al., 2017). A systematic review of the results of these studies suggested that perturbation-based reactive balance training may reduce fall rates by 46% in vulnerable populations such as older adults and patients with Parkinson’s disease (Mansfield et al., 2015).

In contrast, proactive balance training protocols involve voluntary initiation of movement with known support surface interactions. During traditional proactive balance training, the balance system is activated (consciously or unconsciously) in anticipation of predicted perturbations (Lesinski et al., 2015b). Examples include reaching balance tasks and walking mobility tasks. Control-based proactive balance training adds the use of technologies that provide external feedback related to position to aid with movement control. The subject is directed to use the real-time information (e.g., a visual display of center of pressure) to achieve a predefined goal (e.g., steadiness, symmetry, or stability), and thus learns how to associate internal feedback with external cues (Nichols, 1997). An added benefit is that game-design elements and principles can be applied to the interactive components, making the training more fun and engaging (Sihvonen et al., 2004; Szturm et al., 2011).

Both reactive and proactive balance training approaches appear to be useful for both rehabilitation and prevention. However, methodologic limitations and high variability in assessment methods and training parameters preclude making strong statements on the efficacy of these protocols (Zech et al., 2010). In addition, the optimal dosage for balance training has not been fully resolved to date. Based on meta-analyses of dose-response relationships, the most effective balance training interventions to improve steady-state balance require a minimum of 3–6 training sessions per week and at least a total of 16–19 sessions, with each session lasting 11–15 min and each exercise set lasting 21–40 s (Lesinski et al., 2015a). Effective programs also incorporate progressions that increase the difficulty (e.g., use a more unstable surface) or change the type of balance activity (e.g., add dynamic movements) to make the tasks more challenging over time and avoid ceiling effects (DiStefano et al., 2009; Melzer and Oddsson, 2013). The resulting balance improvements are even greater when balance training is combined with resistance training than improvements from balance or resistance training alone (Wolfson et al., 1996; Hirsch et al., 2003; Joshua et al., 2014). One could similarly predict that the addition of balance training to exercise protocols during spaceflight would further attenuate decrements in post-flight balance control.

## Balance Training in Microgravity

We recognize from the history of in-flight exercise hardware that simply adapting Earth-based concepts to a microgravity environment is not always optimal or even feasible (Korth, 2015). For example, axial loading in microgravity results in abnormal postures. On Earth, upright posture is organized by a limited combination of joint angles and body segment positions. The absence of these longitudinal constraints in microgravity results in altered postural strategies when astronauts attempt to stand upright with mediolateral bungees at their hips pulling their feet down to a stable platform (Clement et al., 1984; Clement and Lestienne, 1988). During early adaptation to microgravity, astronauts demonstrate an exaggerated forward tilt posture with greater than normal flexion at the ankles, knees, and hips. A corresponding redistribution of tonic muscle activity is observed between the flexor and extensor muscles of the ankle. In addition, during unexpected forward translation of the foot support in microgravity, the stretch reflex at the ankle joint triggers a burst of activity in the Tibialis anterior, which vanishes after only a few trials (Clement et al., 1985). After several days, astronauts still demonstrate a forward postural tilt, albeit one that is more aligned with a hypothetical gravity vector (Lestienne and Gurfinkel, 1988). Interventions that aim to train upright posture thus need to provide cues for increased multi-joint extension to ensure that simulated postural challenges are compatible with a gravitational environment.

### Recent Observations in Parabolic Flight

**FIGURE 1:**
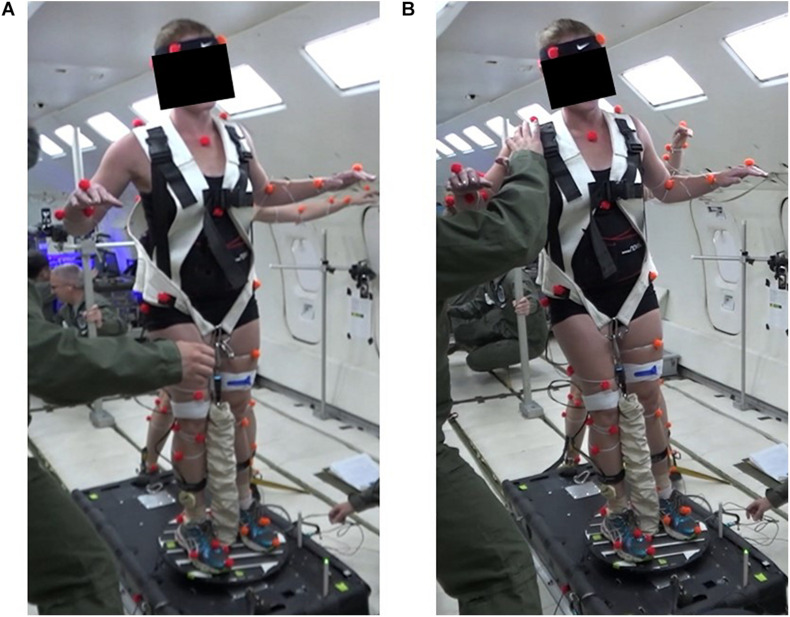
Subject stable on a wobble board in parabolic flight with a single central pull-down load and eyes closed. At the beginning of the 20 s microgravity period **(A)**, the subject begins leaning to the right **(B)** but verbally indicates the perception of leaning to the left (note that this is still a stable position despite the lateral lean). Such orientation illusions commonly occur on entry into weightlessness, depending on the available sensory information (Lackner and DiZio, 1993). Without vision, tactile cues dominate perceived orientation. For example, blindfolded subjects strapped tightly in a seat by a lap belt generally feel inverted, suspended from the belt, during weightlessness (Lackner and DiZio, 2000). Similarly, our subject’s erroneous perception of lean direction with eyes closed is attributed to the interpretation of foot and harness tactile cues.

Our recent observations during parabolic flight further suggest that unstable conditions on Earth are not necessarily unstable in microgravity. During the weightless phases of parabolic flight, subjects were loaded axially onto a platform and instructed to balance on various wobble boards. A similar platform system has been used in parabolic flight previously (Riva et al., 2009). The wobble boards were tested in combination with three different loading configurations: one central load, two lateral loads, or one anterior load and one posterior load. Subjects noted that getting into a neutral starting position was difficult. The axis of rotation of the wobble board needed to be directly beneath their body and, more importantly, directly between the loading attachments. Once positioned on a wobble board, subjects found it easy to maintain balance. However, when they closed their eyes there was often a disconnect between their perceived body lean and the actual direction of their postural tilt (Figure 1). In addition, subjects tended to not react to perturbations because there was no risk of falling (note that Figure 1B is a stable position in microgravity despite the degree to which the subject is leaning). These observations are consistent with previous reports of orientation illusions during postural control in parabolic flight (Lackner and DiZio, 2000). Altered proprioceptive inputs are gradually reinterpreted to eliminate these illusions, meanwhile, individuals adapting to microgravity rely heavily on tactile input for orientation cues, including cues from their feet and the loading harness (Lackner and DiZio, 1993).

**FIGURE 2:**
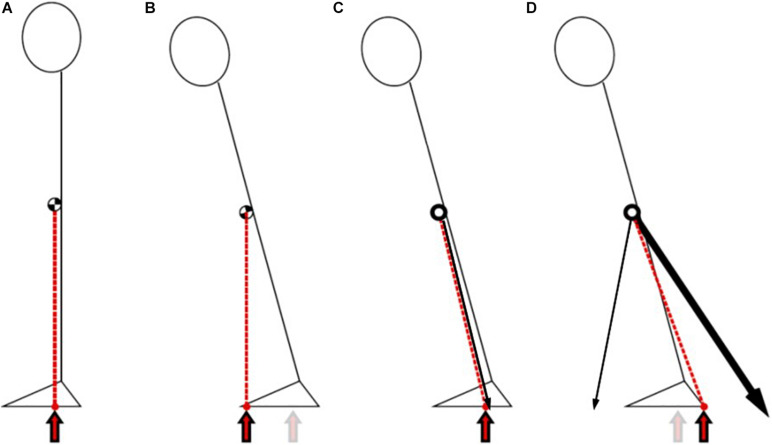
Lateral views of upright bipedal stance on Earth versus simulated standing via bungee loading attachments (black arrows) in parabolic flight. **(A)** Stable upright standing in 1G: center of pressure (COP, red arrow) is essentially a vertical projection of the center of mass (COM, circle at approximately mid-height). **(B)** Leaning forward in 1G: COP must move with COM (relationship shown by red dotted line), toward the limits of stability over the base of support (represented by the triangle feet) to prevent a fall. **(C)** Leaning forward in microgravity with a single central pull-down load or two lateral loads: COP does not move with COM, but instead is anchored by the loading attachment. **(D)** Leaning forward in microgravity with one anterior load and one posterior load: Again, COP does not move with COM, but instead shifts back toward the extended posterior loading attachment while the anterior attachment becomes slack. Note that both **(C)** and **(D)** are stable conditions in microgravity, and do not require subjects to make postural adjustments to prevent a fall.

These observations during bouts of weightlessness lasting 20–25 s suggest that current harness loading attachments do not create unstable postures for subjects during simulated standing. Figure 2 illustrates how the center of pressure (COP, the instantaneous location of the vertical ground reaction force) shifts in relation to the center of mass (COM) during upright posture on Earth versus simulated posture in microgravity. When standing on Earth, the COP reflects the net torque that controls COM position. The horizontal acceleration of COM is proportional to the difference between COP and COM (Winter, 1995). An important biomechanical constraint on balance involves controlling COM within an individual’s limits of stability—the area over which they can move their COM and maintain equilibrium by altering COP without changing their base of support (Horak, 2006). However, in microgravity, the harness attachments that provide axial loading also dictate COP. Because the attachments are fixed, horizontal COM movements are countered by opposing horizontal forces from the harness, such that COM can move outside the normal limits of stability. This lack of instability inhibits the use of reactive responses to remain upright. It is unclear whether the absence of reactive responses could lead to negative effects on return to Earth or whether it could be partially mitigated by a sliding load attachment that follows horizontal COM movements. Overall, the type and minimum amount of balance training required for astronauts to achieve successful post-flight postural outcomes remains unknown. To devise novel approaches, it is essential to understand the underlying factors contributing to unloading-induced balance impairments.

## Proprioceptive and Tactile Contributions to Unloading-Induced Balance Impairments

On Earth, humans are accustomed to using reliable visual, vestibular, and somatosensory input to control complex balance processes (Horak, 2006). For somatosensory input, the CNS receives afferent information from proprioceptors (Golgi tendon organs and muscle spindles) and tactile mechanoreceptors (Ruffini, Merkel, Meissner’s, and Pacinian corpuscles) for conscious and unconscious perception of body position and movement and integrates these signals with visual and vestibular inputs (Duysens et al., 2000; Riemann and Lephart, 2002). The proprioceptive and tactile systems can detect even the slightest body sway during unperturbed upright standing, making them the primary inputs for controlling steady-state posture (Fitzpatrick and McCloskey, 1994). Animal studies support these essential roles. For example, decerebrated cats without vestibular or supraspinal input can maintain balance during treadmill walking (Musienko et al., 2012). Although the lack of vestibular information results in slower and less consistent postural adjustments, these findings indicate that posture and gait control can be accomplished using somatosensory information alone. In contrast, gait is severely compromised in animal models when proprioceptors are removed (Akay, 2020). Therefore, maintaining proprioceptive function should be a priority for protecting postural and locomotor function.

Microgravity alters proprioceptive function, and this appears to affect the feedback control of movement during spaceflight (Watt, 1997). For example, astronauts in orbit have delayed movement strategies and impaired mass discrimination while accelerating a ball up and down using whole-arm movements (Ross et al., 1984; Berger et al., 1997). This may be related to modulations of proprioceptive reflexes. In parabolic flight, vibration-induced proprioceptive illusions are diminished during free fall phases and enhanced during 1.8G phases (Lackner and DiZio, 1992). Thus, gravitational force may modulate the proprioceptive afferent output through descending vestibular modulations of alpha and beta motor neurons. Other proprioceptive illusions occur while hopping after return from spaceflight. During flight, astronauts feel a vertical translation rather than a fall when being “dropped” (i.e., pulled down to the deck), but after flight they perceive the floor being pushed down under them as they hop up and perceive the floor moving up toward them as they hop down (Watt et al., 1985). This is congruent with reduced post-flight postural equilibrium during sway-referenced support surface conditions (Black et al., 1995), a technique used to disrupt proprioceptive feedback. Together, these findings suggest that post-flight postural ataxia is partially mediated by impaired proprioceptive feedback.

Microgravity also alters tactile function essential for maintaining the postural and locomotor control systems. For example, the absence of tactile input from the feet in microgravity alters preparatory activations of the lower limbs that normally precede arm raises on Earth (Layne et al., 1998). The addition of foot pressure via special boots can restore these preparatory activations in microgravity. Because preparatory activations provide necessary stability against the anticipated perturbations of self-movement, maintaining these activations may aid in performance of more complex tasks after flight. In addition, exposure to microgravity alters post-flight foot skin sensitivity. After short-duration spaceflight, astronauts’ feet have increased sensitivity to high-frequency vibrations (250 Hz), and this hypersensitivity is associated with greater reductions in postural equilibrium during sway-referenced support (Lowrey et al., 2014). In contrast, astronauts’ feet are less sensitive to low-frequency vibrations (3 Hz) after spaceflight, and this is associated with attenuated declines in postural stability (Strzalkowski et al., 2015). These altered sensitivities to low- and high-frequency vibrations are thought to be a compensatory strategy related to the redistribution of support loads toward the front of the feet during walking (Saveko et al., 2020). Such a reorganization of support loads may extend the dynamic stabilization phase of walking to aid performance in the early post-flight period while normal postural reactions are lacking. Together, these studies collectively suggest that the foot is an important balance organ, and changes in tactile input from the feet have a significant effect on post-flight balance control.

Microgravity also induces vestibular adaptations that lead to unreliable vestibular inputs during the early post-flight period (Clément and Reschke, 2010). The anticipated detection of translations but not tilt in microgravity becomes maladaptive after return to Earth’s gravitational environment (Parker et al., 1985), causing astronauts to experience sensory discordance, or conflict between the expected and actual sensory input during dynamic activities. Compensatory strategies must be used for functional performance until vestibular recalibration is achieved (Peterka, 2002). One early strategy the CNS uses to respond to unreliable vestibular input is up-weighting more reliable non-vestibular information, like proprioception (Carriot et al., 2015). A pilot study showed that Shuttle astronauts demonstrated transient reweighting of somatosensory cues associated with microgravity-induced vestibular deficiencies (Ozdemir et al., 2018). In this study, postural stability was tested under various eyes-closed conditions, dynamic head tilts were used to disrupt vestibular inputs, and a sway-referenced support surface was used to compromise the reliability of somatosensory inputs. Before spaceflight, the addition of dynamic head tilts negligibly affected postural stability on a fixed support surface, but further destabilized postural stability on a sway-referenced support surface. The ratio between conditions suggests that the availability of reliable somatosensory cues may compensate for disrupted vestibular inputs. This same ratio was exacerbated in astronauts on R + 0 (i.e., there was a greater difference in postural stability between conditions), suggesting less accurate somatosensory utilization associated with spaceflight. Despite these inaccuracies, the vestibular deficiencies were even greater (i.e., vestibular inputs were even more unreliable), resulting in a relative increase in reliance on somatosensory inputs (Ozdemir et al., 2018). Therefore, keeping the proprioceptive system tuned to respond to upright balance challenges in gravitational environments may improve post-flight postural control.

## Proprioceptive Countermeasure Objectives

Countermeasures are intended to maintain sufficient physiological function in all crewmembers, allowing them to perform mission-specific tasks, both nominal and off-nominal, including those immediately after landing, without approaching the limits of their physical capacity (Scott et al., 2019). It is especially challenging to mitigate deficits in post-flight sensorimotor function because interindividual responses are highly variable (Seidler et al., 2015; Wood et al., 2015). During post-flight testing with eyes closed, crewmembers commonly report being unaware of where their feet are. Similar comments are made by HDBR subjects. As one top-performing multi-time flyer explained after displaying minimal post-flight balance decrements, “I just learned to ignore the (unreliable) vestibular input. I still feel it, but I don’t pay attention to it” (personal communication). Whether fellow astronauts or HDBR subjects can be trained to do the same has not been tested.

Since the balance system as a whole cannot be trained in microgravity, the approach presented here involves targeting the proprioceptive and tactile systems that are required for successful balance outcomes. Proprioceptive training is defined as an intervention that targets the improvement of proprioceptive function, focusing on the use of somatosensory signals such as proprioceptive or tactile afferents (Aman et al., 2015). For both healthy adults and clinical populations such as stroke patients, proprioceptive training is a viable method for improving sensorimotor function. Because motor control is inherently multisensory, other forms of training, like exercise, also have a proprioceptive component. In fact, regular physical activity is recommended to attenuate age-related declines in proprioceptive function (Henry and Baudry, 2019). However, as mentioned above, the current aerobic and resistance exercise protocols performed during spaceflight and HDBR are not sufficient to fully mitigate post-flight and post-bed rest decrements in balance control (Miller et al., 2018; Mulavara et al., 2018). Given that exercise provides body loading and stimulates proprioceptive and tactile afferents, it is likely that these inputs must be delivered in the context of balance control challenges if they are to maintain post-flight postural stability (Riva et al., 2009). This concept suggests that proprioceptive and tactile inputs be used, and not just present, to increase their value for generating appropriate motor output (Bakkum et al., 2020).

At the mechanistic level, an in-flight proprioceptive countermeasure may keep the proprioceptive system tuned to respond to upright balance challenges in gravitational environments. This approach takes advantage of the convergence of multisensory cues to aid improvement in performance during periods of sensory discordance due to gravitational transitions (Carriot et al., 2015; Ozdemir et al., 2018). By keeping both visual and proprioceptive inputs veridical, astronauts may be able to ignore unreliable vestibular input until their sensorimotor state is reorganized appropriately for the gravitational environment. This dynamic re-weighting of multimodal inputs may also facilitate vestibular recalibration (Toth et al., 2019). Thus, in addition to aiding functional abilities for emergency situations immediately upon landing, an effective countermeasure may accelerate performance recovery on standard tasks that certify an astronaut’s readiness for scheduled EVAs.

## Promising Modalities for an In-Flight Proprioceptive Countermeasure

Various potential countermeasures have been proposed. Unlike candidates for systemic benefits to neuromuscular function and neuronal health, like nutraceuticals (Kumar and Sharma, 2010; Mortreux et al., 2019) or enhanced radiation shielding (Parihar et al., 2018; Blackwell et al., 2020), many training countermeasures appear to target similar specific mechanisms. Thus, we present evidence supporting the inclusion of four promising modalities in an in-flight proprioceptive countermeasure: axial body loading, postural/proprioceptive challenges, tactile input, and sensory feedback. For comprehensiveness, we also outline the feasibility of electromagnetic and electrical stimulation techniques. This approach is intended to help generate new ideas for the integration or enhancement of potential countermeasures. The modalities may be used in different combinations, and an optimal solution might target multiple. Each of these modalities can be adapted for HDBR models to help accelerate testing and development.

### Axial Body Loading

Body load sensing is important for controlling dynamic balance function because it modulates motor control (Dietz and Duysens, 2000). Even acute unloading appears to decrease ankle proprioception with corresponding decreases in lower leg muscle activation (Marchant et al., 2020). Altering body load perception via water immersion decreases electromyographic activity, maximal voluntary contractions, and spinal reflexes in the antigravity extensors (Pöyhönen and Avela, 2002). In contrast, adding loads out of the water does not elicit the opposite effect, likely because load afferents are saturated with input (Dietz et al., 1989). Similar results have been found using a body support harness. After only 30 min of walking with 40% body weight support, subjects exhibit acute alterations in various gait parameters and increased vestibular-mediated compensatory head movements during normal treadmill walking (Mulavara et al., 2012). Thus, body load sensing plays an integral role in locomotor control and modulates the central interpretation of vestibular input.

Countermeasures should aim to provide axial loading up to one full-body weight to stimulate load afferents. Various methods of loading have been used. A wearable garment called the Penguin Suit has been used in-flight to provide chronic axial loading throughout the day and during treadmill running (Kozlovskaya and Grigoriev, 2004). Although the Penguin Suit can attenuate soleus muscle fiber atrophy during HDBR (Ohira et al., 1999), cosmonauts refused to wear it in-flight due to significant thermal and movement discomforts (Waldie and Newman, 2011). A similar concept called the Gravity Loading Countermeasure Skinsuit (GLCS) was designed to improve comfort and loading characteristics. Although promising test results suggest that the GLCS may be beneficial as an adjunct to exercise (Attias et al., 2017; Carvil et al., 2017), the efficacy of this loading strategy for postural and locomotor control has not been directly tested in a randomized controlled trial. The axial loading by itself may not be sufficient to protect against post-flight balance impairments.

Earlier we described how greater resistance training loads provided by the ARED attenuates post-flight decrements in agility and postural stability compared to its predecessor (Wood et al., 2011). However, the effects are not fully protective. Likewise, although a harness and bungee cord loading system is used on the ISS with the T2, the benefits of loading itself have not been tested. A more integrative approach to loading like lower body negative pressure (LBNP) can simultaneously introduce mechanical loading and foot-ward fluid shifts (Hargens et al., 1991). For example, there is some evidence to suggest that treadmill exercise within LBNP can mitigate balance impairments after 30 days of body unloading via HDBR (Macaulay et al., 2016). Although the additional foot-ward fluid shifts may not directly mitigate post-flight balance impairments, LBNP may have added indirect benefits through the mitigation of cardiovascular risks during spaceflight (Petersen et al., 2019; Harris et al., 2020). For example, LBNP may attenuate spaceflight-associated neuro-ocular syndrome, which appears to increase visual dependence during sensorimotor tasks (Lee et al., 2019).

### Postural/Proprioceptive Challenges

Proprioception is closely linked to movement. Given this and the training principle of specificity (Wolfson et al., 1996; Hirsch et al., 2003; Joshua et al., 2014), it is essential that proprioceptive countermeasures incorporate the sensory and motor aspects of the desired outcome (Aman et al., 2015). Since there are no falls in microgravity, reactive training (i.e., generating motor responses to sensory input) is not feasible, but proactive training (i.e., initiating motor output to achieve an external target and consequential sensory input) is. Exercise data suggest that proprioceptive countermeasures without postural challenges may have limited effects on balance. For example, although resistance training has an inherent proprioceptive component, resistance training alone during HDBR has no observable effect on post-bed rest balance impairments (Haines, 1974; Kouzaki et al., 2007). This reinforces the idea that balance deficits are associated with decrements in proprioceptive utilization, rather than insufficient muscle function. Thus, successful in-flight/in-bed countermeasures might need to mimic upright standing on Earth, including the need to quickly engage proprioceptive sensing mechanisms and activate secondary stabilizer muscles used in terrestrial balance control processes (Nichols, 1997; Mansfield et al., 2015). These secondary stabilizer muscles have an essential role in postural control contributing to joint stiffness, but may not receive the same training stimulus as prime mover agonists during resistance training in microgravity.

Another method of providing proprioceptive challenges involves shifting the control point from the body to an external environment with inherent instability. For example, the lower extremity dexterity task uses a spring-loaded support surface to test the capability to regulate dynamic interactions between the foot and support (Lyle et al., 2013). Performance on this task is thought to represent maximal sensorimotor abilities at submaximal force levels and is strongly associated with age, sex, and athletic ability (Lawrence et al., 2014, 2017). However, the efficacy of using such a task in the form of training is unclear. Intervention trials are needed to determine the efficacy of training and the transferability of learned skills to functional task performance. Regardless of the type of proprioceptive challenges employed, adding task variability and progressively raising the difficulty of challenges may further facilitate sensorimotor re-adaptation to gravitational environments by increasing the generalization of motor skills to untrained movements (Bloomberg et al., 2015; Bakkum et al., 2020).

### Tactile Input

It has been hypothesized that the loss of support afferentation experienced during spaceflight induces neuromuscular dysfunction leading to loss of tonic muscle activation and subsequent post-flight postural and locomotor instability (Kozlovskaya et al., 2007). To restore the absent neuromuscular activation, researchers have tested various in-flight dynamic foot stimulation techniques. Investigators found that mechanical stimulation via pressurized boots restores neuromuscular activation throughout the entirety of the lower limb musculature and leads to preserved lower limb strength and locomotor function (Layne and Forth, 2008). Similar effects of mechanical stimulation have been demonstrated during DI (Ogneva et al., 2011; Zakirova et al., 2015). Importantly, the prevention of DI-induced structural and functional changes in soleus fibers were observed only with mechanical foot stimulation and not with electrical muscle stimulation, suggesting that tactile input is more important than contractile activity for protection against gravitational unloading. The benefits of applying mechanical stimulation to the feet have also been shown during HDBR (Reschke et al., 2009). Two groups of subjects completed an obstacle course test before and after 60 days of HDBR. The first group served as bed rest controls while the second group received daily foot massages to ameliorate tenderness in the soles of their feet. Both groups took significantly longer to complete the obstacle course after bed rest than before. However, the foot massage group had only a 27% increase in time compared with a 94% increase in time for the non-foot massage group. These data suggest that foot pressure and tactile input are central components in the control of posture and locomotion.

There are various other ways to provide tactile input to the feet in microgravity. A simple way to add tactile stimulation is through textured shoe insoles for use during in-flight aerobic and resistance exercise. This may help improve active joint position sense at the ankle during loading like has been demonstrated on Earth (Waddington and Adams, 2000, 2003). Whether the enhanced stimulation can also improve proprioceptive function over time (Steinberg et al., 2017, 2019), and thus mitigate declines during the mission, requires further investigation. A proprioceptive countermeasure that axially loads astronauts toward their feet up to the equivalent of one-full body weight can simultaneously provide proprioceptive and tactile input within a balance-challenge context. One concept is to instruct astronauts to control their body orientation during simulated postural tasks while being loaded on a moveable platform under their feet (English et al., 2020a). The downward force that provides body support loading will be translated through the feet for full-body segmental coordination and platform tilt manipulation. Both anterior-posterior and medial-lateral platform tilts are important. Medial-lateral displacements are thought to be more destabilizing than anterior-posterior displacements for the active control of balance in posture and gait (Oddsson et al., 2007). However, HDBR studies have commonly observed greater postural sway disturbances in the anterior-posterior direction (Dupui et al., 1992; Viguier et al., 2009; Muir et al., 2011). This is likely due to increased demand for lower-limb coordination combined with atrophy in the distal muscles responsible for control. In addition, intrinsic trunk stiffness and damping are smaller in the anterior and posterior directions than the lateral directions (Vette et al., 2014), thus demanding greater sensory feedback-based control. Providing both axial body loading and tactile input on a moveable platform may activate the postural stabilizing muscles in a coordinated fashion that simulates multidirectional sway on Earth.

### Sensory Feedback

Proprioceptive training can be enhanced by real-time, non-proprioceptive feedback (Aman et al., 2015). Adding sensory cues via auditory, tactile, or visual modalities provides relevant information about posture that can aid performance and the interpretation of proprioceptive input (Sienko et al., 2018; Henry and Baudry, 2019). Incorporating visual feedback will be especially important in microgravity to ensure that simulated postural challenges are compatible with a gravitational environment. A visual COP display can help astronauts align their body orientation along an axial force vector, perpendicular to the support surface (Riva et al., 2009), and facilitate their COP movements near Earth-based limits of stability. Visual feedback also allows the introduction of game design concepts that inform astronauts of their performance, track their progress, and increase engagement (Szturm et al., 2011). This can also be provided in the form of virtual reality (VR) to expand sensory experiences during proprioceptive training. A vast literature demonstrates that the CNS requires a rich and varied sensory environment to maintain normal structure and function (Sale et al., 2014). In animal studies, sensory environmental enrichment has been shown to provide a CNS protective effect against radiation (Fan et al., 2007) and promote social and behavioral adaptability in response to changing environmental conditions (Gelfo, 2019; Gubert and Hannan, 2019). Even short but highly rewarding interactions with a stimulating context can induce positive effects on brain and behavior comparable to those produced by extended exposures (Rojas-Carvajal et al., 2020). Thus, VR could augment proprioceptive training by providing an interesting virtual environment that will keep crewmembers cognitively engaged and challenged during long-duration exploration class missions (Wu et al., 2016).

Vibrotactile feedback may also improve sensorimotor performance and training by providing an intuitive orientation reference without distracting users from natural visual cues (Lawson et al., 2016). This approach uses touch response as cues, often accomplished through subtle, textured vibrations from electromechanical actuators. For postural control on Earth, a sensor, such as an inertial measurement unit, detects body tilt relative to gravity and modulates vibration intensities accordingly. Such vibrotactile systems have been developed as sensory substitution devices for patients with visual, vestibular, or tactile impairments and to assist users in spatial orientation and navigation in unfamiliar environments. For example, the efficacy of vibrotactile feedback to improve sensorimotor skills has been demonstrated in blind patients (Flores et al., 2015), aviators (Rupert et al., 2016), athletes (van Breda et al., 2017), and astronauts post-spaceflight (Gurfinkel et al., 1993; Clément et al., 2018). As a training tool, vibrotactile stimulation has been shown to augment balance improvements in patients with peripheral neuropathy (Oddsson et al., 2020), multiple sclerosis (Leonard et al., 2017), stroke (Badke et al., 2011), and normal healthy aging (Bao et al., 2018). Although the mechanisms by which vibrotactile stimulation is processed and used by the CNS are not well understood, there is some support in the literature for sensory reweighting or increased reliability of intact sensory channels (Sienko et al., 2018). Vibrotactile feedback may therefore improve in-flight proprioceptive training by increasing the value of proprioceptive inputs for sensorimotor performance. In a previous technology demonstration (van Erp Jan et al., 2007), one ISS astronaut wore a vest with 56 vibrating tactors to present a coordinate reference using localized vibrations on the torso pointing in the “down” direction (toward the control unit on the floor). The vest improved orientation speed and accuracy, and the astronaut reported no issues with respect to the sensation of the tactors, their localizability, or their comfort. Although the potential operational benefits were limited to challenging situations, similar technology may be advantageous for body orientation and tilt awareness during in-flight proprioceptive training.

### Electromagnetic and Electrical Stimulation

Current trends in neurorehabilitation involving muscle and brain stimulation techniques may someday lead to solutions that get integrated in a sensorimotor countermeasure suite. Electrical muscle stimulation (EMS) has well-established positive effects on muscle size and function, especially during disuse and aging (Chae et al., 2008; Kern et al., 2014). In addition, several studies have demonstrated improvements in postural control in older adults as a result of either static or dynamic EMS (Langeard et al., 2017). The physiological stimulus may extend beyond simple muscle contractions, providing a sensory stimulation that further contributes to the contraction (Song et al., 2012). There are, however, limited studies of EMS efficacy during spaceflight or spaceflight analogs. An 8-cahnnel EMS device designed to be worn as trousers was developed for a pilot experiment on the MIR space station (Mayr et al., 1999). Despite showing some potential benefits, this work was not pursued further after initial feasibility testing (Rafolt et al., 2000). Similarly, in small HDBR and DI studies, EMS attenuated declines in muscle size and strength (Duvoisin et al., 1989; Ogneva et al., 2011), but there remains a lack of evidence for the effects on postural and locomotor control. Future studies are needed to evaluate these effects of EMS as a countermeasure during spaceflight or spaceflight analogs.

Brain stimulation offers a promising strategy for restoring the functional state of the sensorimotor system; however, based on recent evidence, the underlying concepts need to be further clarified, and approaches need to be further developed. Most of the positive evidence for brain stimulation involves small enhancements of upper extremity function in clinical populations (Micera et al., 2020). An improved mechanistic understanding is needed to prescribe complex neuromodulation targets like postural control (Labruna et al., 2019; Jonker et al., 2021). In addition, current models used to describe stimulation-based learning (or re-learning after injury) do not support retaining a sensorimotor state over a long-duration mission (Formento et al., 2018; Lozano et al., 2019). Thus, brain stimulation may be better suited for post-flight rehabilitation than in-flight countermeasures. Finally, large resting motor threshold changes have been observed during short periods of microgravity in parabolic flight (Badran et al., 2020). Potential etiologies may include an upward brain shift, increased cortical excitability, changes in intracranial pressure, changes in the peripheral nervous system, or some combination of these. These additional factors will need to be characterized before translating an intervention from ground analogs to flight missions.

## In-Flight Proprioceptive Assessments

Another key consideration for proprioceptive countermeasures is the incorporation of assessment capabilities. In-flight assessments are critical to provide objective quantifications of performance, as it relates to the proprioceptive countermeasure system, to track changes over time and thus monitor training effectiveness (Han et al., 2016). The repeated measurements can be used to personalize training prescriptions and tailor the proprioceptive challenge to each crewmember’s abilities. Note that although these results provide important information within the countermeasure, they may not directly translate to functional task performance post-landing. For example, there could be significant learning effects during spaceflight, resulting in improved performance while the underlying construct of proprioceptive function remains unchanged (i.e., maintained). Therefore, we must differentiate between the utility of assessments for prescription as described above and the utility of assessments for predicting post-flight postural and locomotor control. The later requires a better understanding of the effects of spaceflight on proprioception than currently exists. No measures of proprioceptive function related to postural stability have been performed pre-landing during long duration spaceflight. Thus, determining the efficacy of proprioceptive countermeasures must be based on post-flight postural and locomotor control tasks rather than in-flight proprioceptive assessments. As spaceflight missions venture further from low-Earth orbit and communication between crewmembers and their medical support teams decreases, the importance of establishing predictive utility for proprioceptive countermeasure assessments will likely increase to help inform crew of their readiness to perform critical mission tasks. Given the requirement for a control group and the operational constraints in-flight, this likely must be tested in a long duration spaceflight analog like HDBR or DI.

## Conclusion

NASA’s Human Research Program is in the early stages of development for an in-flight operational countermeasure that supplements aerobic and resistance exercise for the mitigation of post-flight decrements in postural and locomotor control. One approach involves keeping the proprioceptive and tactile systems reliable enough to overcome transient vestibular deficiencies for functional task performance upon return to a gravitational environment. The four promising modalities identified for inclusion in the countermeasure include axial body loading, postural/proprioceptive challenges, tactile input, and sensory feedback. Integrating these modalities with other countermeasures (such as LBNP) may increase the efficiency of risk mitigation strategies and have additional indirect benefits. In terms of training schedule, some data suggest that undulated training approaches utilizing variable postural challenges and conditions of feedback may enhance the benefits for adapting to novel sensorimotor environments (Bloomberg et al., 2015). However, these studies have not been conducted during spaceflight or spaceflight analogs. Therefore, future investigations are warranted to determine the optimal training parameters given individual differences in proprioceptive utilization (Seidler et al., 2015). A successful countermeasure might also translate to ground-based balance training interventions and assessments. Older adults and various clinical populations known to experience declining proprioceptive function may benefit from similar training methods on Earth (Aman et al., 2015; Oddsson et al., 2015). Thus, the development of an in-flight proprioceptive countermeasure may have widespread impact on our understanding of balance control and the mitigation of fall risks.

## Author Contributions

TM, BP, and JB conceived of the presented idea. TM took the lead in writing the manuscript. BP, SW, and JB contributed to the comprehensive examination of the literature. GC and LO provided critical feedback that helped shape the balance training and proprioception countermeasure sections. All authors discussed and contributed to the final manuscript.

## Conflict of Interest

TM, BP, and GC were employed by KBR and LO was employed by RxFunction. The remaining author declares that the research was conducted in the absence of any commercial or financial relationships that could be construed as a potential conflict of interest.
